# Supplementary material for: Handheld Ultrasound or Conventional Ultrasound Devices in Patients Undergoing HCT: A Validation Study
Source: J Clin Med. 2023 Jan 8;12(2):520. doi: 10.3390/jcm12020520 (PMC9867323; doi:10.3390/jcm12020520)
Supplement: Supplementary file 1 [file jcm-12-00520-s001.zip › jcm-2060259-supplementary.pdf]

TABLE S1

RAW DATA OBTAINED IN ULTRASOUND EXAMS

	Mean	Std. Dev.	Std. Error	Count	Minimum	Maximum	# Missing
**Conventional PV diameter, total**	1.081	.200	.022	80	.650	1.800	5
**Conventional PV diameter, -7**	1.019	.148	.037	16	.650	1.300	1
**Conventional PV diameter, 0**	1.115	.154	.038	16	.800	1.400	1
**Conventional PV diameter, +7**	1.088	.247	.062	16	.800	1.800	1
**Conventional PV diameter, +14**	1.116	.250	.062	16	.800	1.800	1
**Conventional PV diameter, +21**	1.070	.184	.046	16	.800	1.500	1
**Handheld PV diameter, total**	1.071	.236	.026	80	.600	2.000	5
**Handheld PV diameter, -7**	1.061	.206	.051	16	.600	1.500	1
**Handheld PV diameter, 0**	1.028	.185	.046	16	.700	1.300	1
**Handheld PV diameter, +7**	1.119	.277	.069	16	.800	1.900	1
**Handheld PV diameter, +14**	1.096	.323	.081	16	.600	2.000	1
**Handheld PV diameter, +21**	1.052	.178	.044	16	.800	1.500	1
**Conventional RHL diameter, total**	15.811	2.497	,279	80	11.000	21.300	5
**Conventional RHL diameter, -7**	15.581	2.696	.674	16	11.000	19.500	1
**Conventional RHL diameter, 0**	15.887	2.321	.580	16	11.100	19.500	1
**Conventional RHL diameter, +7**	15.913	2.991	.748	16	12.000	21.300	1
**Conventional RHL diameter, +14**	15.875	2.253	.563	16	12.500	19.300	1
**Conventional RHL diameter, +21**	15.800	2.468	.617	16	13.000	19.700	1
**Handheld RHL diameter, total**	15.153	2.161	.242	80	11.400	20.000	5
**Handheld RHL diameter, -7**	14.675	2.033	.508	16	11.400	18.500	1
**Handheld RHL diameter, 0**	15.325	1.860	.465	16	12.000	19.000	1
**Handheld RHL diameter, +7**	15.227	2.693	.673	16	12.000	20.000	1
**Handheld RHL diameter, +14**	15.231	2.165	.541	16	12.000	18.700	1
**Handheld RHL diameter, +21**	15.306	2.179	.545	16	11.800	18.300	1
**Conventional LHL diameter, total**	9.348	1.968	.220	80	5.100	13.500	5
**Conventional LHL diameter,** -7	8.975	1.828	.457	16	6.100	12.100	1
**Conventional LHL diameter, 0**	9.156	1.990	.497	16	6.600	12.200	1
**Conventional LHL diameter, +7**	9.194	2.036	.509	16	5.100	12.000	1
**Conventional LHL diameter, +14**	9.581	1.868	.467	16	7.000	13.500	1
**Conventional LHL diameter, +21**	9.831	2.220	.555	16	5.800	12.500	1
**Handheld LHL diameter, total**	9.162	1.771	.198	80	5.300	14.000	5
**Handheld LHL diameter, -7**	8.475	1.611	.403	16	6.000	11.000	1
**Handheld LHL diameter, 0**	9.000	1.747	.437	16	6.700	12.000	1
**Handheld LHL diameter, +7**	9.331	2.050	.512	16	5.300	14.000	1
**Handheld LHL diameter, +14**	9.269	1.623	.406	16	6.500	12.400	1
**Handheld LHL diameter, +21**	9.737	1.763	.441	16	6.110	13.400	1
**Conventional spleen diameter, total**	10.544	4.417	.510	75	5.300	25.000	10
**Conventional spleen diameter, -7**	10.933	5.385	1.390	15	6.000	25.000	2
**Conventional spleen diameter, 0**	10.720	4.527	1.169	15	5.400	21.000	2
**Conventional spleen diameter, +7**	10.747	4.186	1.081	15	6.300	20.000	2
**Conventional spleen diameter, +14**	10.300	3.837	.991	15	5.300	19.500	2
**Conventional spleen diameter, +21**	10.020	4.561	1.178	15	6.000	22.000	2
**Handheld spleen diameter, total**	10.485	4.233	.489	75	6.000	25.000	10
**Handheld spleen diameter, -7**	10.940	5.170	1.335	15	6,500	25.000	2
**Handheld spleen diameter, 0**	10.633	4.329	1.118	15	7.000	20.100	2
**Handheld spleen diameter. +7**	10.627	3.845	.993	15	6.900	19.500	2
**Handheld spleen diameter, +14**	10.277	3.942	1.018	15	6.000	19.000	2
**Handheld spleen diameter, +21**	9.947	4.278	1.104	15	6.000	20.500	2

File S1. Bland-Altman's Plot for each patient

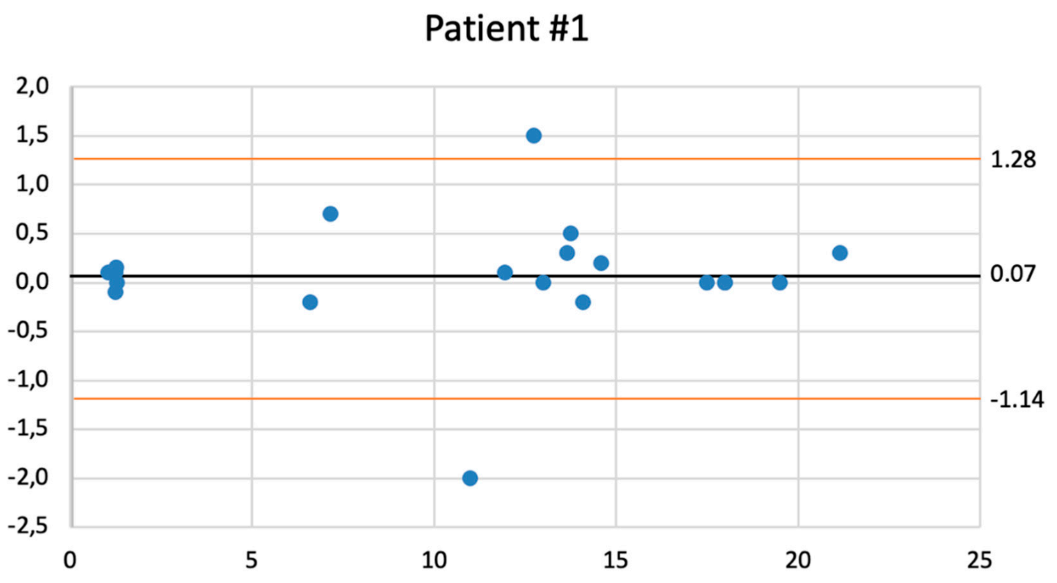

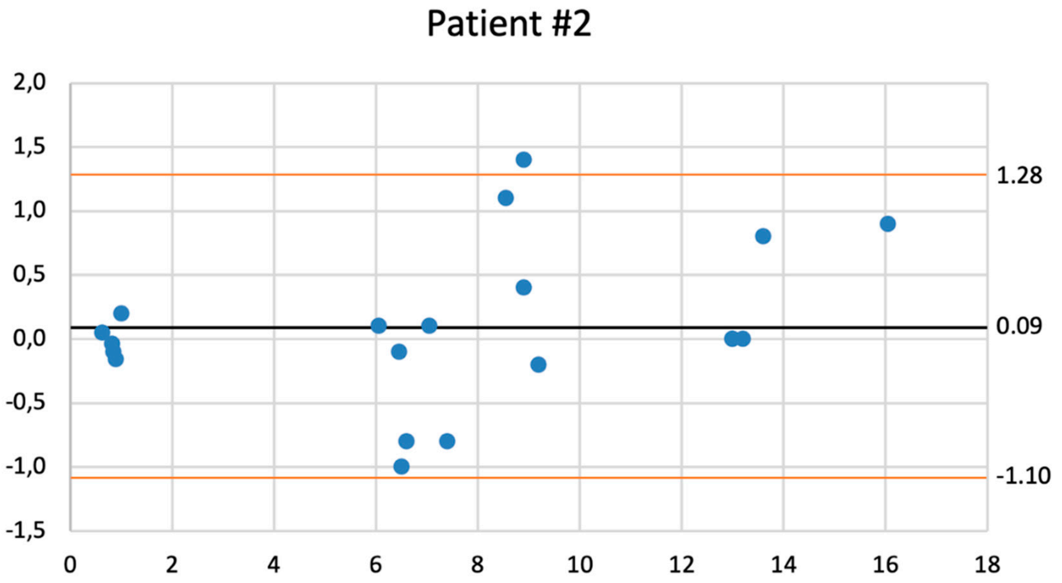

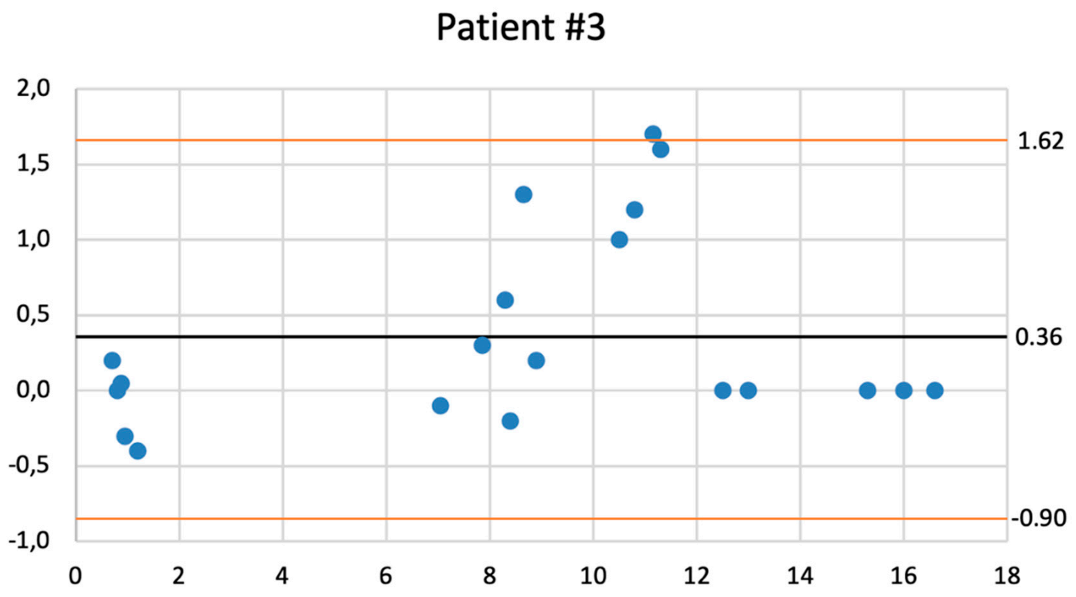

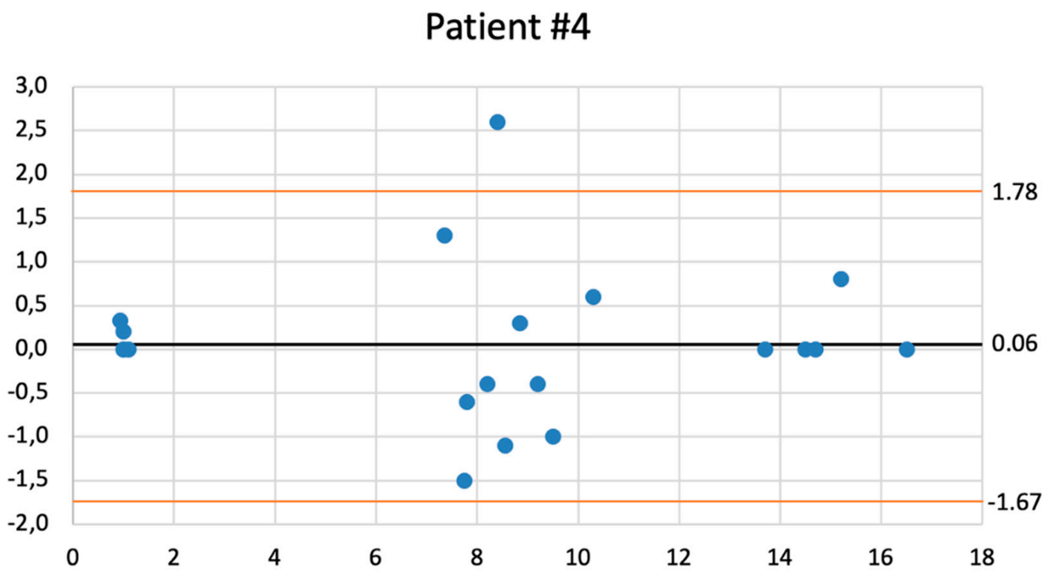
Patient #4
3,0
2,5
2,0
1,5
1,0
0,5
0,0
-0,5
-1,0
-1,5
-2,0
0
2
4
6
8
10
12
14
16
18
1.78
0.06
-1.67

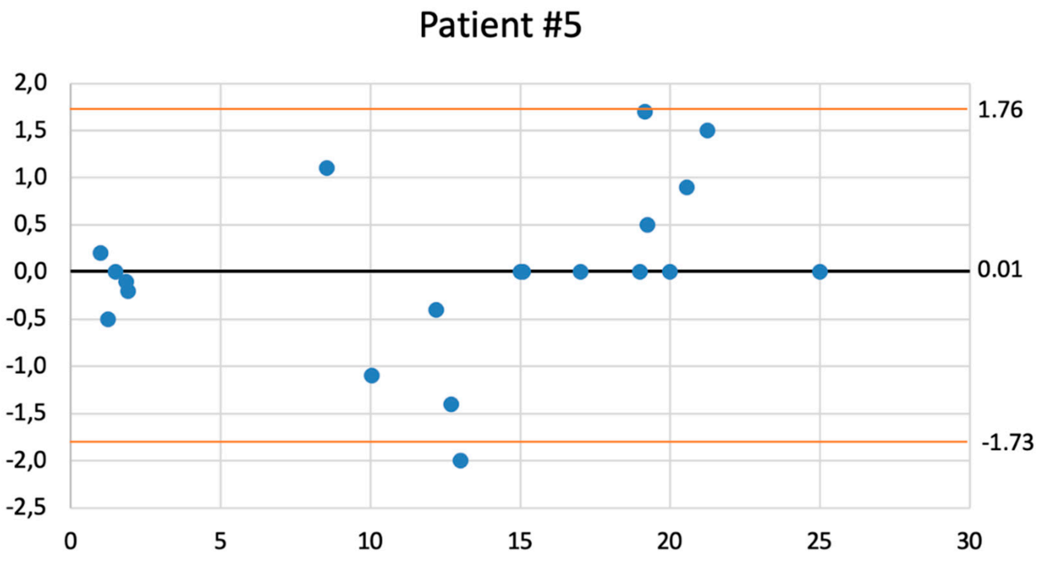
Patient #5
2,0
1,5
1,0
0,5
0,0
-0,5
-1,0
-1,5
-2,0
-2,5
0
5
10
15
20
25
30
1.76
0.01
-1.73

Patient #6

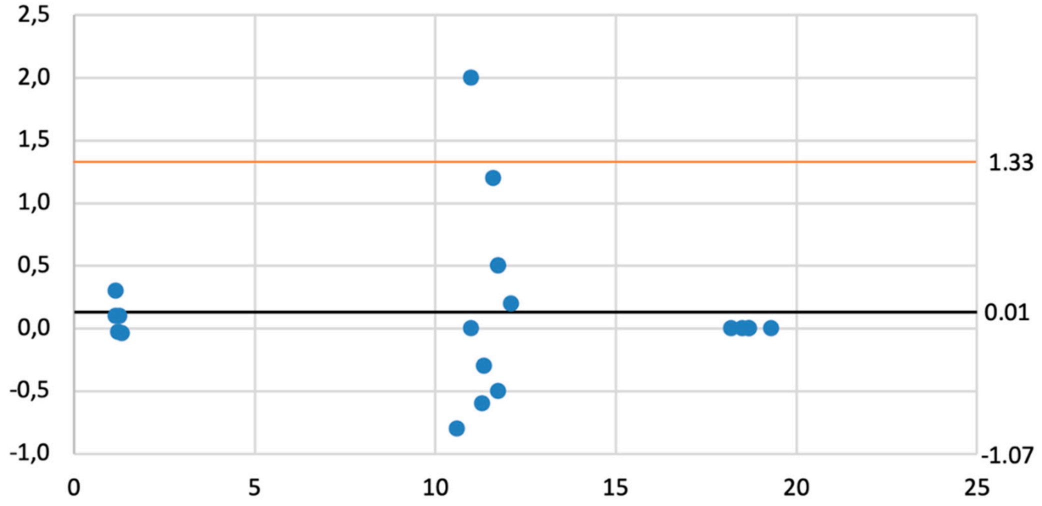

Patient #7

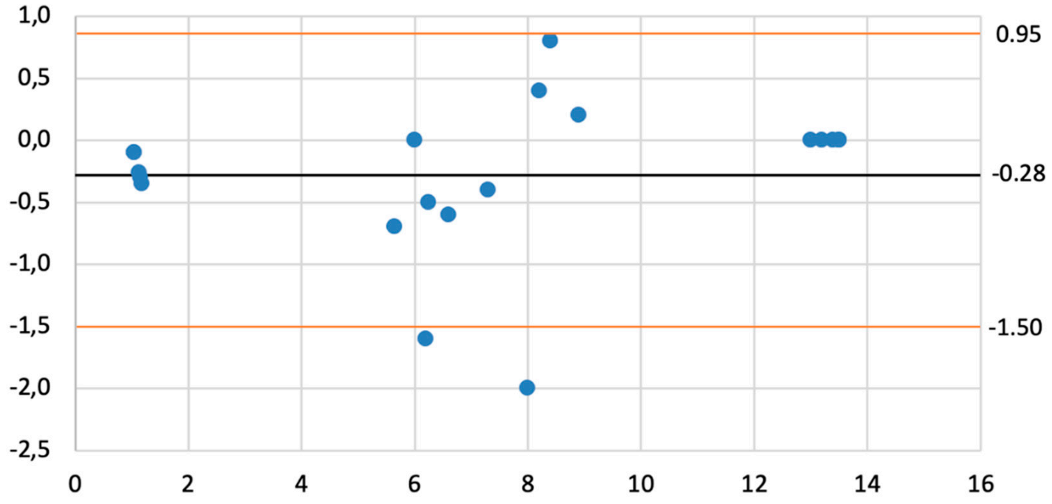

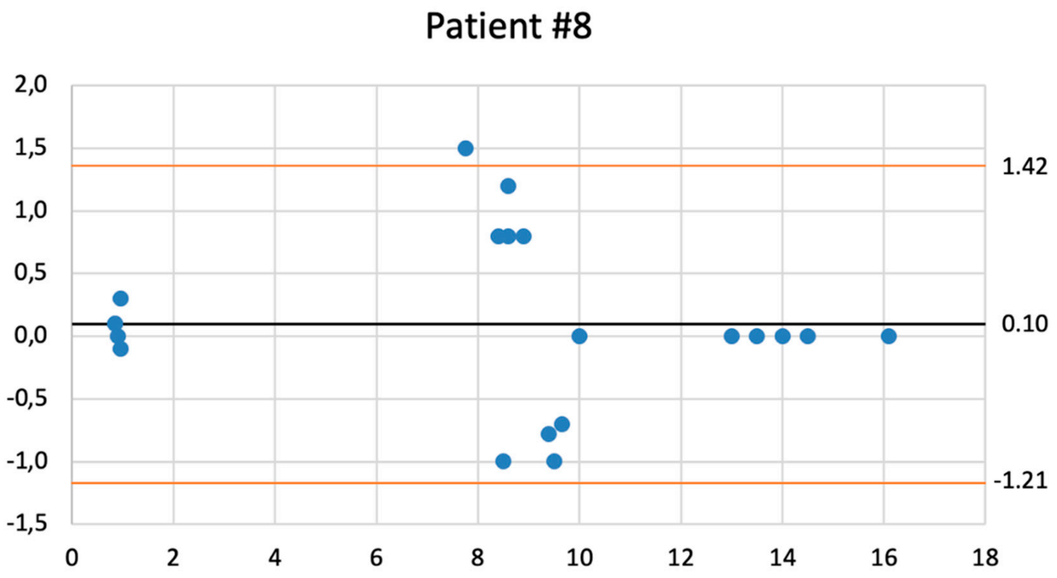
Patient #8
2,0
1,5
1,0
0,5
0,0
-0,5
-1,0
-1,5
0
2
4
6
8
10
12
14
16
18
1.42
0.10
-1.21

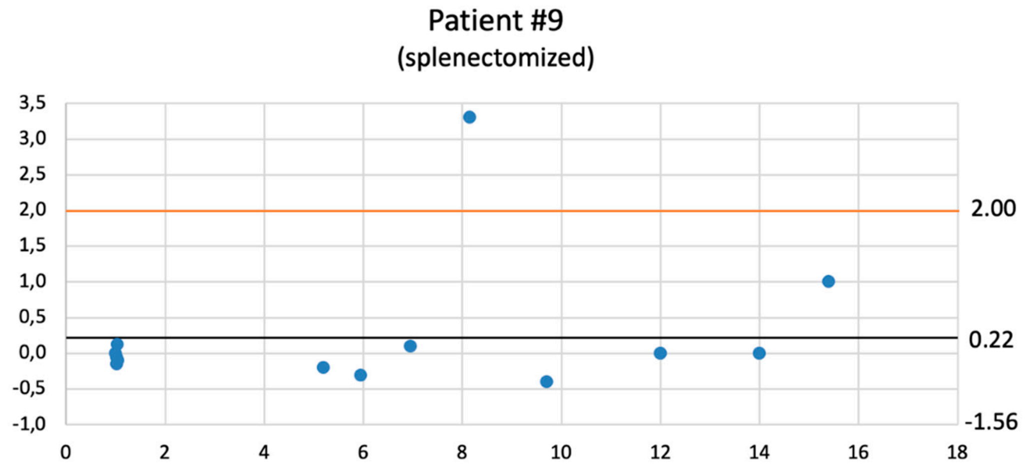
Patient #9
(splenectomized)
3,5
3,0
2,5
2,0
1,5
1,0
0,5
0,0
-0,5
-1,0
0
2
4
6
8
10
12
14
16
18
2.00
0.22
-1.56

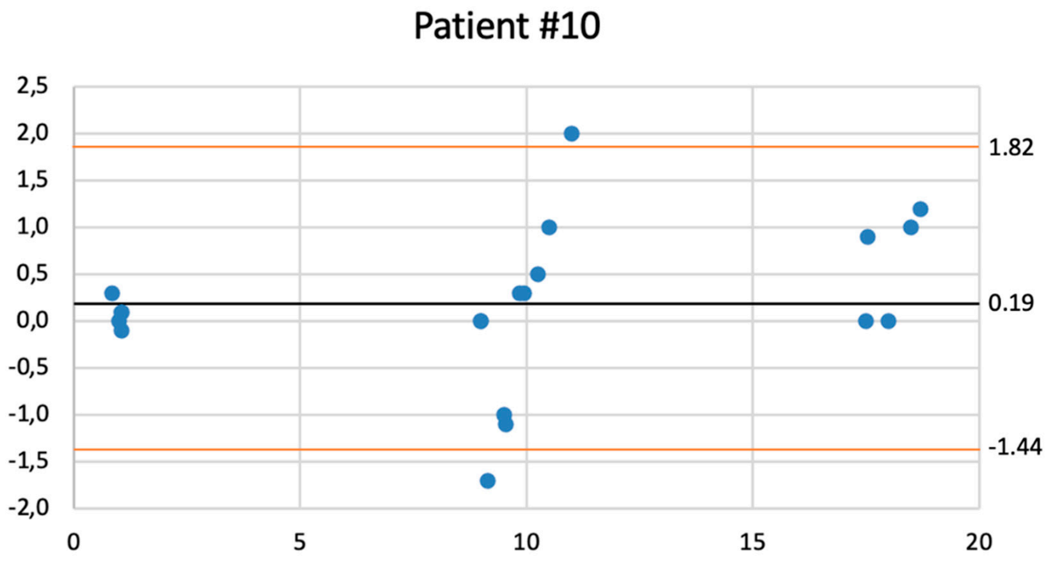
Patient #10
2,5
2,0
1,5
1,0
0,5
0,0
-0,5
-1,0
-1,5
-2,0
0
5
10
15
20
1.82
0.19
-1.44

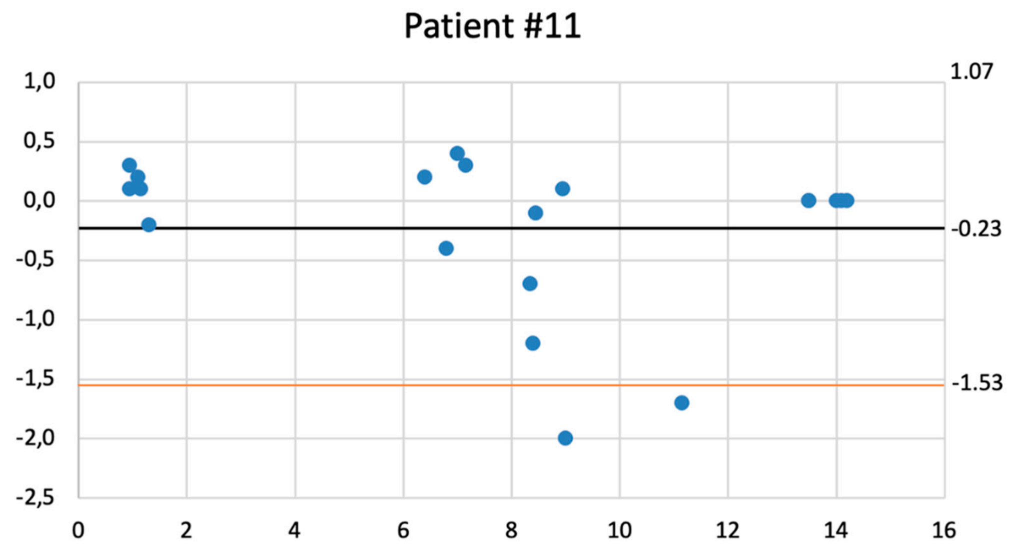

Patient #11
1,0
0,5
0,0
-0,5
-1,0
-1,5
-2,0
-2,5
0
2
4
6
8
10
12
14
16
1.07
-0.23
-1.53

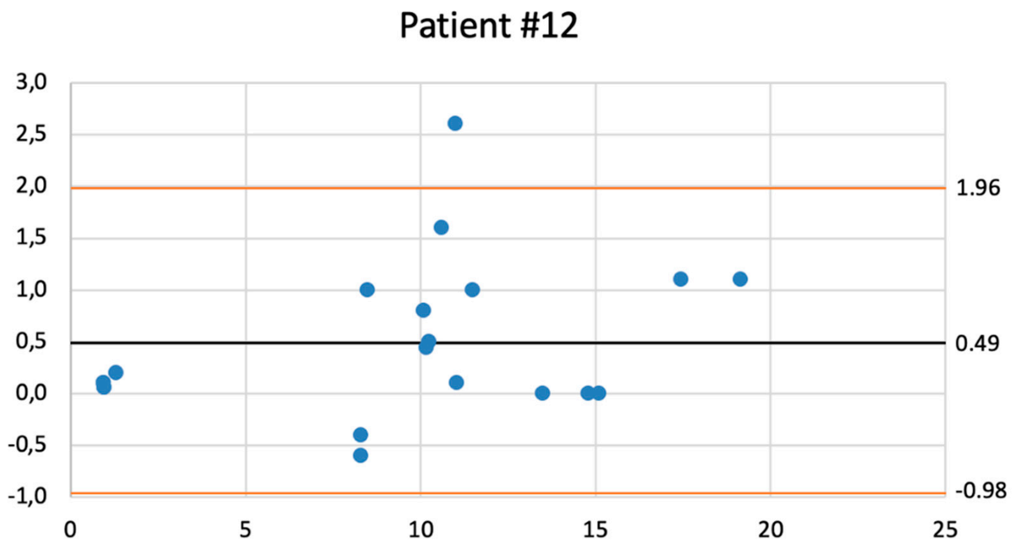

Patient #12
3,0
2,5
2,0
1,5
1,0
0,5
0,0
-0,5
-1,0
0
5
10
15
20
25
1.96
0.49
-0.98

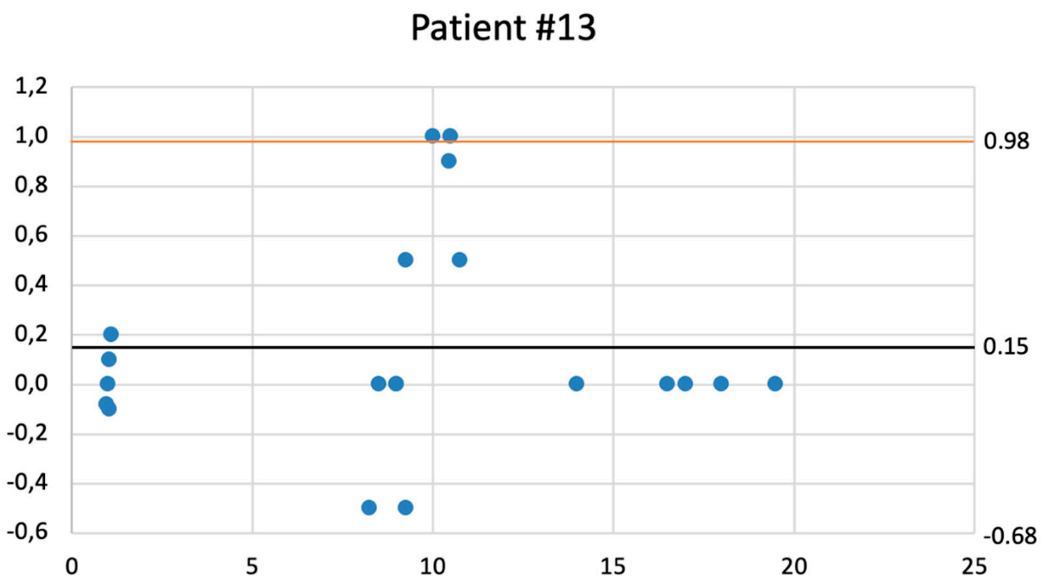
Patient #13
1,2
1,0
0,8
0,6
0,4
0,2
0,0
-0,2
-0,4
-0,6
0
5
10
15
20
25
0.98
0.15
-0.68

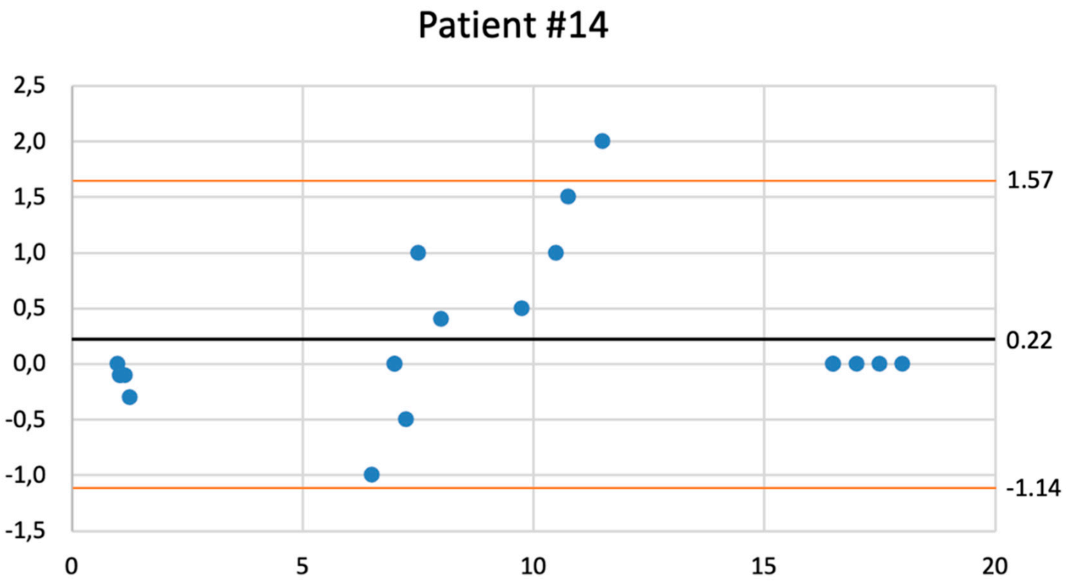
Patient #14
2,5
2,0
1,5
1,0
0,5
0,0
-0,5
-1,0
-1,5
0
5
10
15
20
1.57
0.22
-1.14

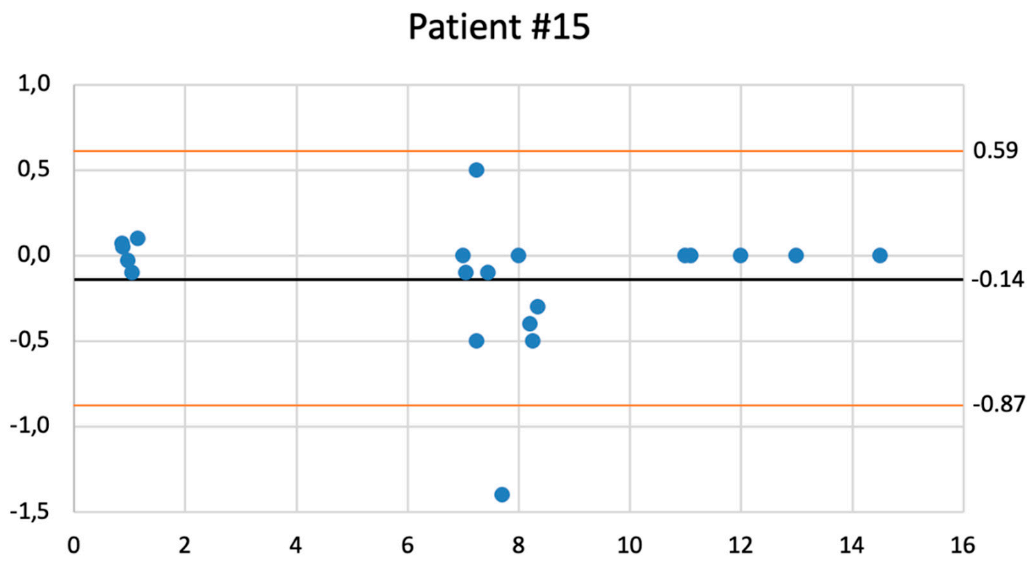
Patient #15
1,0
0,5
0,0
-0,5
-1,0
-1,5
0
2
4
6
8
10
12
14
16
0.59
-0.14
-0.87

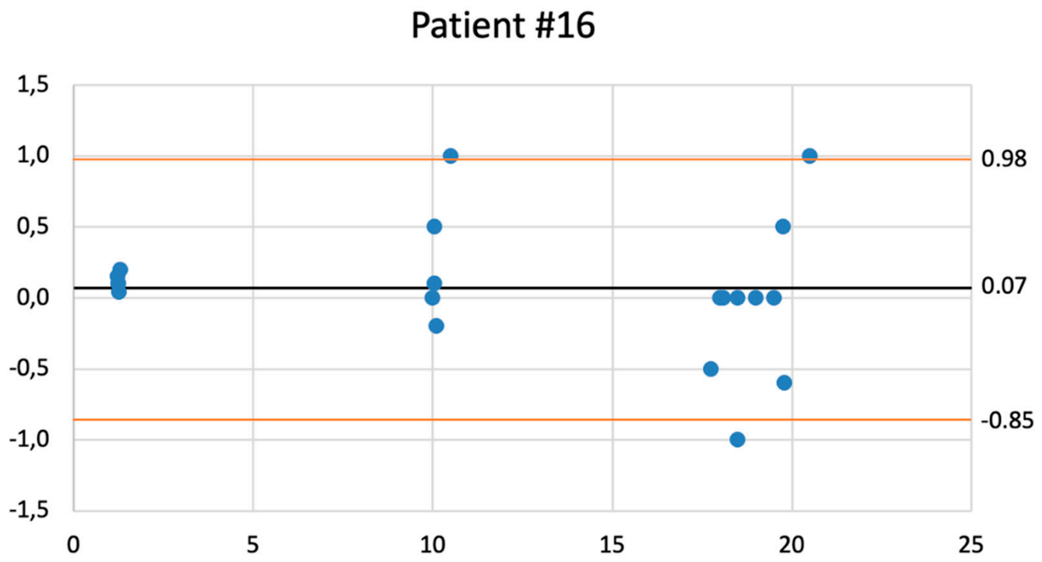
Patient #16
1,5
1,0
0,5
0,0
-0,5
-1,0
-1,5
0
5
10
15
20
25
0.98
0.07
-0.85
